# Molecular Characteristics of Barley Yellow Dwarf Virus—PAS—The Main Causal Agent of Barley Yellow Dwarf Disease in Poland

**DOI:** 10.3390/plants12193488

**Published:** 2023-10-06

**Authors:** Katarzyna Trzmiel, Beata Hasiów-Jaroszewska

**Affiliations:** Department of Virology and Bacteriology, Institute of Plant Protection—National Research Institute, 60-318 Poznań, Poland; b.hasiow@iorpib.poznan.pl

**Keywords:** BYDV-PAS, RT-PCR, RFLP, CP, RdRp, genetic variability

## Abstract

Barley yellow dwarf is a threat to cereal crops worldwide. Barley yellow dwarf virus—PAS (BYDV-PAS) was detected for the first time in Poland in 2015, then in 2019. In the spring of 2021, in several locations in Poland, winter wheat and barley plants with dwarfism and leaf yellowing were collected. Reverse transcription—polymerase chain reaction results revealed BYDV presence in 47 samples and excluded wheat streak mosaic virus infections. Next, immuno-captured polymerase chain reactions confirmed only one case of co-infection caused by BYDV and wheat dwarf virus. Moreover, restriction fragment length polymorphism analysis showed that BYDV-PAS was predominant. The preliminary results were confirmed using sequencing. Infected cereal plants originated mainly from northwestern Poland. The complete coding sequence of coat protein (CP) and a fragment of RNA-dependent RNA polymerase (RdRp) genes of 14 Polish isolates were determined and deposited in the GenBank database. The nucleotide and deduced amino acid sequences of local isolates were compared with others reported to date, indicating their high similarity, from 75.4% to 99.5% and from 81.1% to 100% nucleotide sequence identity, in RdRp and CP, respectively. Phylogenetic analysis, based on the CP gene, revealed the presence of 3 main groups. The Polish isolates clustered together within the Ia group.

## 1. Introduction

Barley yellow dwarf (BYD) is one of the most important viral diseases of cereal crops in terms of both widespread occurrence and economic significance. In the past, several outbreaks of this disease were observed in various regions of the world: in 1907 and 1949 in the USA, in 1976 and 1986 in Canada, in 1977 in Italy, in 1998 in China, in 2003 in Spain, in 2002 and 2008 in the Czech Republic, and in 2001/2002 in Poland [1]. BYD is induced using a group of spherical barley yellow dwarf viruses (BYDVs) transmitted by about 25 aphid species [2,3]. The first classification of BYDVs distinguished different viruses by their primary aphid vector [4,5] or based on their serological properties [6] and next on nucleic acid sequences [7,8,9]. Currently, barley yellow dwarf virus species (BYDV)-kerII, -kerIII, -MAV, -PAS, and -PAV are grouped as the genus *Luteovirus*; cereal yellow dwarf virus species (CYDV)-RPV, -RPS and maize yellow dwarf virus-RMV (MYDV-RMV) are classified into the genus *Polerovirus* and BYDV-GAV, -SGV and -GPV are unassigned members in the family Luteoviridae [10]. The BYDV has a positive sense single-stranded (+ss) RNA genome of 5.6–5.8 kb in length with no 5′- cap and no poly(A) tail, and it is composed of 6 open reading frames (ORFs) [11]. In the first step of luteoviruses infection, their genomic RNA (gRNA), which encodes p1-helicase (ORF1) and p1-p2—RNA dependent RNA polymerase (RdRp), is translated. RdRp is created using a ribosomal frameshift of ORF1 and ORF2. Next, due to the polymerase activity of RdRp, replication of gRNA and transcription of sub-genomic RNAs (sgRNA1, sgRNA2, and sgRNA3) occur. The sgRNA1 serves as a template for the synthesis of p3a (ORF3a), p3—major component of coat protein (CP) (ORF3), p4—movement protein (MP) (ORF4) and p3-p5—minor component of CP (ORF3 and ORF5). The minor CP is expressed as an ORF 3/5 fusion by suppression of the ORF3 stop codon, expressing additional RTD protein (50 kDa) and producing larger (72–80 kDa) read-through protein (CP-RTD) [1]. In this study, two fragments, OFR2 and ORF3, will be analyzed. Viral infection causes leaf discoloration, yellowing, and dwarfism of infected plants. BYD severity depends on three main determinants: virus access time, virus species type, and cultivar genotype [1]. The disease is especially harmful for winter forms of cereals. Autumn infection caused by BYDVs can reduce grain yield by 63% and spring infection by 41% [12], with an average of 30% [13]. Although yield losses usually increase with the increase in disease incidence, BYD can reduce the yield even if the plant’s infection is asymptomatic [14]. On the other hand, in field conditions, plants are often infected by more than one virus species. As it has been previously shown, mixed infection of BYDV-PAV with wheat dwarf virus (WDV), wheat streak mosaic virus (WSMV), or barley mild mosaic virus (BaMMV) can affect the symptoms and disease severity [15]. Historical reports revealed local infections caused most often by single or mixed infections of BYDV-PAV and BYDV-MAV, and rarely of CYDV-RPV, in small-grain, in Poland [16]. However, further studies revealed the presence of new species (BYDV-PAS, BYDV-SGV, and BYDV-GAV) and numerous cases of co-infection [17,18,19,20].

The aim of this study was to determine the type of infection as well as evaluate the genetic variability of newly collected BYDV isolates and comparison with others described to date. 

## 2. Results

### 2.1. Molecular Diagnostics

**Figure 1:**
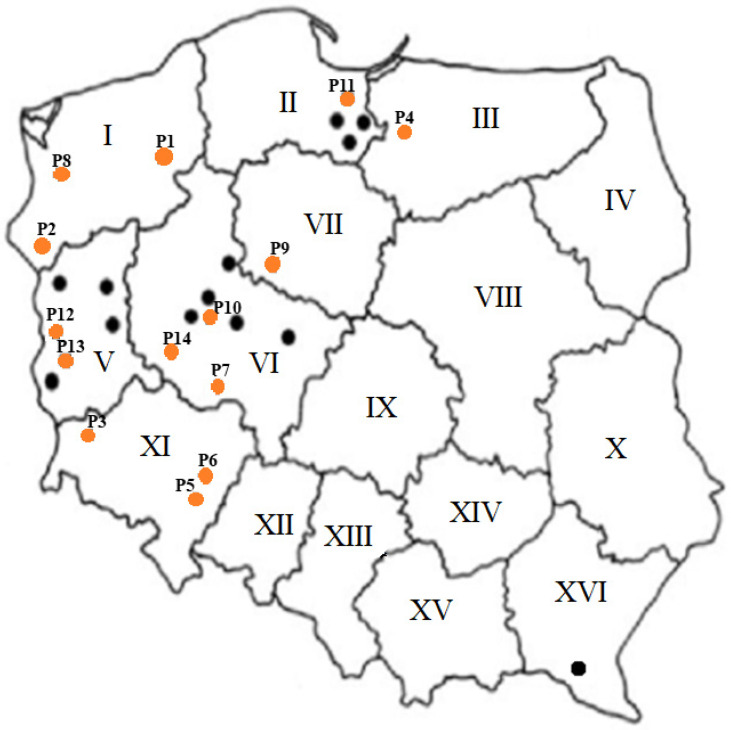
Locations of BYDV-PAS infections in Poland in Spring 2021. Black dots represent the locations of BYDV-PAS infection, orange dots show the locations where the samples were collected for sequencing. Names of the regions: I—West-Pomerania, II—Pomerania, III—Warmia-Masuria, IV—podlaskie, V—Lubusz, VI—Greater Poland, VII—Kujavia-Pomerania, VIII—Masovia, IX—łódzkie, X—lubelskie, XI—Lower Silesia, XII—opolskie, XIII—Silesia, XIV—świętokrzyskie, XV—Lesser Poland, XVI—Subcarpathia.

**Figure 2:**
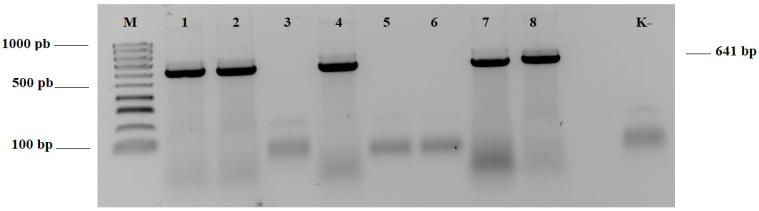
Detection of BYDV coat protein (CP) region (641 bp) in plant samples by RT-PCR. Amplified products were analyzed in 1% TBE agarose gel. Lanes: (M)—100 bp DNA ladder (Novazym); (1−5)—tested wheat sample; (6−8)—tested barley samples; (K−—no template control.

**Figure 3:**
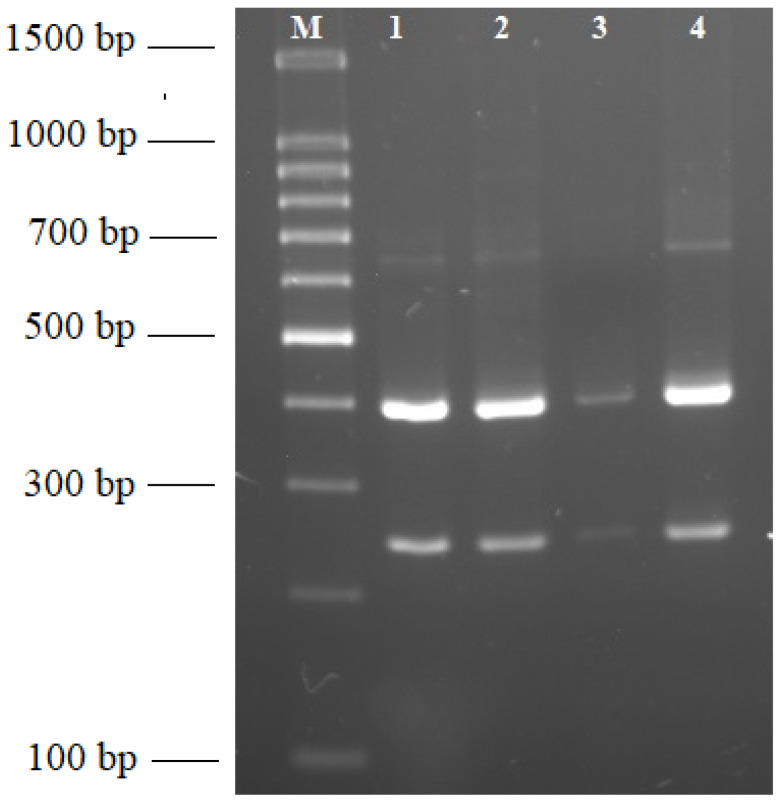
Restriction profiles of BYDV species. RT-PCR products were digested by Hpa II endonuclease and separated on 2% TBE agarose gel. Lanes: (M)—100 bp DNA ladder (Novazym); (1–4)—tested BYDV samples.

The results of this study confirmed virus presence in the majority of analyzed plant samples (47 positive/50 tested) collected from 20 various locations, mostly in northwestern Poland (Figure 1). The symptoms of 3 virus-free samples (2 wheat and 1 barley) from one location in the Greater Poland region have probably been caused by other pathogens, e.g., fungi or other factors affecting cereals. Molecular diagnostic results showed the prevalence of BYDV in tested samples. Partial results of RT-PCR tests are presented in Figure 2. The RFLP analyses revealed one characteristic banding pattern after digestion of the RT-PCR products by Hpa II, which indicates BYDV-PAS infections (Figure 3). In IC-Duplex-PCR tests, the presence of WDV-B specific amplicons (483 bp in size) was obtained only for three tested plant samples from one location in the Lubusz region. Moreover, additional RT-PCR tests with WSMV-specific primers did not confirm plant infection. The sequencing results of the obtained RT-PCR products were consistent with the results of the RT-PCR-RLFP reaction and confirmed BYDV-PAS presence in all analyzed samples.

### 2.2. Comparative Analysis

**Figure 4:**
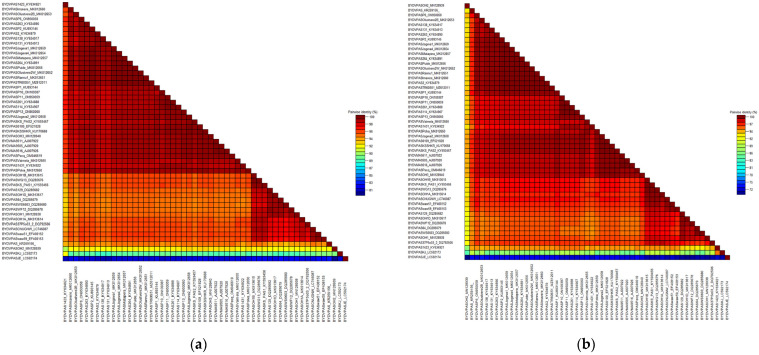
Two-dimensional visualization of nucleotide sequence identity (**a**) or amino acid sequence identity (**b**) of 46 barley yellow dwarf virus—PAS (BYDV-PAS) isolates of CP gene. The matrices were performed using SDTv1.2.

**Figure 5:**
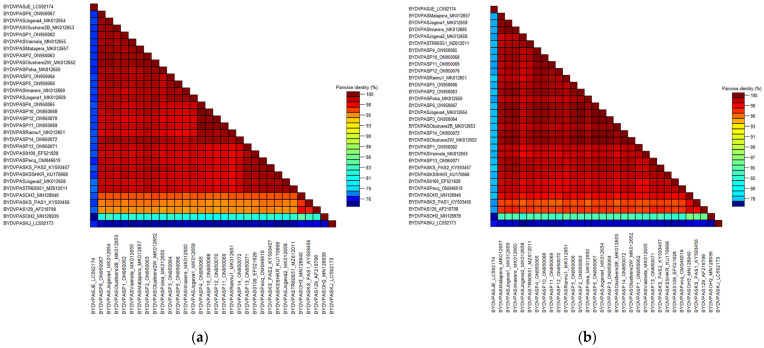
Two-dimensional visualization of nucleotide sequence identity (**a**) or amino acid sequence identity (**b**) of 30 isolates of barley yellow dwarf virus—PAS (BYDV-PAS) of RdRp gene. The matrices were performed using SDTv1.2.

Obtained results allowed us to determine the complete coding sequence of CP (603 nucleotide, nt) as well as the partial coding sequence of RdRp (942 nt) genes of the Polish BYDV-PAS isolates. A set of nucleotide sequences (from BYDV-PAS-P1 to BYDV-PAS-P14) were deposited in the NCBI GenBank database. Their short characteristics with the accession numbers of individual samples are presented in Table 1. Comparative analysis of CP nt sequences revealed 100% identity for BYDV-PAS -P2, -P3, -P4, -P5, -P7, -P8, -P9, and -P12, as well as for BYDV-PAS-P11 and -P14 isolates. For the remaining Polish samples, some variability in nt and amino acid (aa) sequences was observed, and their identity ranged from 98.8% to 99.8% and from 97.2% to 100%, respectively. Analogically, the homological RdRp partial coding sequence was revealed only for four isolates (BYDV-PAS -P4, -P7, -P8, and -P9). The alignment results for the other Polish isolates showed that nt and aa sequence identity ranged from 97.5% to 99.8% and from 98% to 100%, respectively. The results indicated the dominance and prevalence of one genetic variant in various regions of the country, as well as a slight variation in obtained coding sequences of CP and RdRp. Four out of the 14 obtained sequences of RdRp and CP shared 100% identity. Interestingly, these isolates originated from various host plants (wheat and barley) growing in different parts of the country during different seasons (2014/2015 and 2020/2021). Comparison of CP nt sequences in the analyzed BYDV population revealed identity ranging from 81.1% to 100% and from 72.6% to 100% for aa sequences, respectively. The analysis of RdRp sequences showed identity ranging from 75.4% to 99.5% and from 78% to 100% for nt and aa, respectively. The dominant variant within the Polish population (BYDV-PAS-P2) shared 100% identity with the group of Estonian isolates (-Jogeva1, -Jogeva4, -Matapera, -Puide, -Rannu1, -Olustvere 2-W), German isolates (BYDV-PAS-26-1, -26-4, -114) as well as to Turkish one (BYDV-PAS-TR60-S51). Moreover, 100% identity of aa sequences of obtained RdRp fragment of BYDV-PAS-P2, -P3, -P6, -P14 to -Jogeva4 and -Olustvere2-W was also demonstrated. Nevertheless, the analyzed group of BYDV-PAS isolates shared the slightest identity with both South Korean isolates BYDV-PAS-JE and -KJ, originating from infected oat plants. For CP, the range of identity was 81.2–81.8% and 89.2–89.6% (nt sequences) and 72.2–73.3% and 85–86.1% (aa sequences), for BYDV-PAS-JE and -KJ, respectively. Analogically, for RdRp, nt sequence identity values ranged from 78.3% to 78.9% and from 75.4 to 76%, and for the aa sequence, from 83.4% to 84.1% and from 78% to 79%, respectively. The visualization of nt and aa sequence’s identity for CP and RdRp fragment, performed by SDTv1.2, was shown in Figure 4 and Figure 5, respectively.

### 2.3. Assessment of Variability and Recombination Analysis

Detailed analysis assessed the variability of aa sequences in two hypervariable regions of CP. The first region encompasses 45–60 aa residues (A/S/TGRRGPN/DSI/VPGSA/TGRT), the second one 155–168 aa residues (EAINGKD/EFQESTID). Both of them have been used for the differentiation of BYDV species [21]. The results of multi-sequence alignment analysis of the first region of CP showed a division into three main groups of isolates: (i) Polish, Estonian, German, Moroccan, Belgium, Turkish and some part of American (BYDV-PAS-0109, -OH3, -KS-SHKR, KS-PAS2); (ii) the most part of American isolates, (iii) Pakistani, New Zealand and South Korean (BYDV-PAS-CNU-GNW, from wheat). Moreover, a clear difference and the presence of up to 10 aa changes in this CP region was demonstrated for the remaining South Korean isolates BYDV-PAS-KJ and -JE, originating from oat plants. Analogically, for the second CP region, more conservative within BYDV-PAS population, the presence of two main groups was revealed: (i) Polish, Estonian, German, Moroccan, Belgium, and some part of American (BYDV-PAS-0109, -OH3, -KS-SHKR, KS-PAS2); (ii) the rest part of American isolates, Pakistani, New Zealand, Turkish and South Korean (BYDV-PAS- CNU-GNW, -KJ). BYDV-PAS-JE, like in the mentioned region, shared only four common aa residues and differed from the other BYDV-PAS isolates in 12 of 16 positions. The recombination analysis performed for the RdRp fragment revealed the existence of one potential recombinant—South Korean BYDV-PAS-JE, with potential major BYDV-PAS-OH3 and minor BYDV-PAS-KJ parents. Analogical analysis for CP pointed to German BYDV-PAS-143-2 isolate with major BYDV-PAS-Olustvere2-B and minor BYDV-PAS-KJ parents. The putative recombinants were detected using at least 6 RDP4 detection methods; therefore, they were not considered in the next phylogenetic analysis.

### 2.4. Phylogenetic Analysis

**Figure 6:**
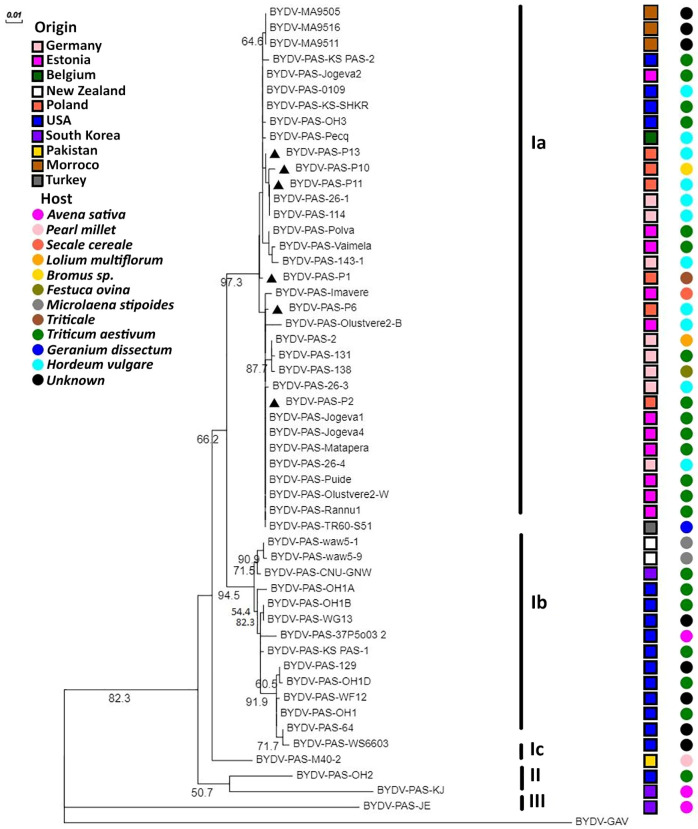
Maximum likelihood tree based on 540 nt fragment of CP region of BYDV-PAS isolates. The nucleotide sequence of BYDV-GAV was used as an outgroup. The numbers of each major node indicate bootstrap values out of 1000 replicates (provided only when >50%). The phylogenetic tree was edited and visualized in the Evolview online platform. Information about the host and region of origin was placed on the tree and explained in the legend. Polish BYDV-PAS isolates are indicated (black triangles).

**Figure 7:**
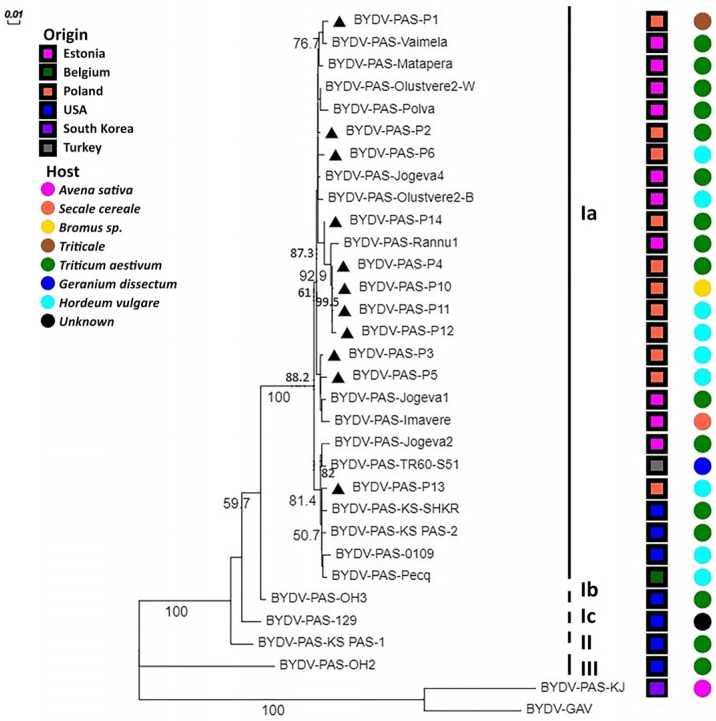
Maximum likelihood tree based on 942 nt fragment of RdRp region of BYDV-PAS isolates. The nucleotide sequence of BYDV-GAV was used as an outgroup. The numbers of each major node indicate bootstrap values out of 1000 replicates (provided only when >50%). The phylogenetic tree was edited and visualized in the Evolview online platform. Information about the host and region of origin was placed on the tree and explained in the legend. Polish BYDV-PAS isolates are indicated (black triangles).

The phylogenetic analysis showed the presence of 3 main clusters. The first one, which was the most variable, was divided into three subgroups, named Ia, Ib, and Ic. The results performed for two various regions of the BYDV-PAS genome (CP and RdRp gene fragments) were convergent (Figure 6 and Figure 7). 

In general, a similar arrangement of generated phylogenetic trees was observed, but slight differences were also noticeable. In the tree based on CP nt sequences, all the Polish isolates are grouped in the same cluster (Ia). However, closer phylogenetic relationships between BYDV-PAS-P10, -P11, and -P13 and some distance between BYDV-PAS-P1, -P2, and -P6 were also noticed. The group of Polish BYDV-PAS isolates clustered together with Estonian, German, Moroccan, and four isolates from the USA. The American isolates, BYDV-PAS-KS-SHKR, -KS-PAS-2, -0109, and -OH3, originated from Kansas, Iowa, and Ohio, respectively (Table 1). Moreover, single isolates from Belgium (BYDV-PAS-Pecq) and Turkey (BYDV-PAS-TR60-S51) were also included in this cluster. The second subgroup, Ib, was created mostly by different American isolates, with two isolates from New Zealand (BYDV-PAS-waw5-1 and -waw5-9) and one originating from Republic of Korea (BYDV-PAS-CNU-GNW). The last one, Ic, was represented by only a single, more distinct Pakistani BYDV-PAS-M40-2 isolate from *Pennisetum glaucum* (pearl millet). The second cluster was created using only two isolates, BYDV-PAS-OH2 from Ohio and BYDV-PAS-KJ from Republic of Korea. The third cluster, and one separate branch, was represented by the South Korean isolate BYDV-PAS-JE. For the RdRp tree, the available nt sequences came from Poland, Estonia, the USA, Republic of Korea, Belgium, and Turkey. Phylogenetic analysis based on the nt sequence of RdRp gene fragment confirmed the similar location of BYDV-PAS-P1, -P2, and -P6 isolates, but in this variant, stronger differentiation and location of BYDV-PAS-P13 on another branch of Ia was revealed. Moreover, the American isolate (BYDV-PAS-OH3) belonging to the Ia subgroup of the CP tree in the RdRp tree was classified as a separate Ib subgroup. On the other hand, the next American isolates (BYDV-PAS-129 and -KS-PAS1), clustered together in the Ib of the CP tree, were divided into two separate branches, Ic and II subgroups, respectively. Finally, BYDV-PAS-KJ, clustered together with BYDV-PAS-OH2 in the II group of the CP tree, in the RdRp tree, was merged with BYDV-GAV, used as an outgroup. Similar results were obtained based on aa sequence of both analyzed regions. For CP, the Polish BYDV-PAS isolates shared from 98.8% to 100% nt identity with a group of European and Morrocan isolates, while with American, New Zealand, and Pakistani from 95.5% to 96.2%, respectively. Analogically, for RdRp, the corresponding values were 97.5–99.8% and 92.8–99.5%.

## 3. Discussion

Knowledge about the presence and diversity of BYDV species in local fields is very important in the epidemiology context for two main reasons: firstly, the severity of the symptoms can depend on the particular BYDV/CYDV/MYDV species; secondly, the transmission efficiency of virus species varies with the aphid vector species [22]. In addition, as it has been previously reported [23], virus prevalence varies from year to year, so this kind of information may be useful for disease management in the future. 

BYDV-PAS was separated from BYDV-PAV, considered a distinct species, and placed on the ICTV list in 2002 [9] due to genome sequence divergences. Current species demarcation criteria within the Luteoviridae family take into account >10% differences in aa sequences of any viral gene product [24]. BYDV-PAS has been confirmed in many countries around the world: the USA [25], New Zealand [26], Republic of Korea [27], Morocco [28], and in Europe—France [29], Czech Republic [30], Poland [18], Estonia [31]. However, the real BYDV-PAS occurrence might be underestimated. According to Chay et al. [32], BYDV-PAV antiserum does not discriminate between BYDV-PAV and BYDV-PAS. In addition, even based on sequencing results, some BYDV-PAS samples have been wrongly classified as BYDV-PAV [25]. Finally, the existence of recombination events complicates proper classification and the taxonomy of BYDV species [31].

The published results, based on serological methods, indicated the dominance of BYDV-PAV and -MAV with a lower proportion of CYDV-RPV infections in Poland [16]. However, further molecular analysis of cereal samples from the last epidemic BYD in the 2014/2015 season revealed the presence of other species: BYDV-PAS, -SGV, and -GAV in the country. Moreover, mixed infection of BYDV-MAV/WDV, BYDV-PAV/WDV, and BYDV-MAV/-PAV/-PAS occurred commonly and led to synergistic effects [18]. Further research from 2019 confirmed the co-infection of BYDV-MAV/BYDV-PAS and BYDV-MAV/BYDV-PAV and revealed the spread of newly detected species to other regions of the country. Furthermore, the obtained results showed a new tendency for the common occurrence of BYDV-PAS and BYDV-SGV in the group of BYDV species detected in cereal crops in Poland [19]. Current research results revealed increasing dominance of single BYDV-PAS infection with only one case of BYDV-PAS/WDV-B co-infection. Based on the above results, it can be assumed that BYDV-PAS is currently the main factor causing BYD symptoms in Poland. These observations are in contrast with those reported by Hodge et al. [33] from the USA (Ohio) and Kim et al. [27] from Republic of Korea, where BYDV-PAV was predominant among BYDV species. On the other hand, our findings are consistent with the results from Europe, the Czech Republic [34,35,36], and Estonia [31], where BYDV-PAS abundance was confirmed both using high-throughput sequencing (HTS) and conventional RT-PCR results. According to Authors from the Czech Republic, prevalent BYDV-PAS distribution might be partly explained by (i) high BYDV-PAS incidence on tested volunteer plants and wild grasses in which it can overwinter, (ii) a wider range of examined aphid vectors (four species), in comparison to BYDV-PAV (2 species) or BYDV-MAV (only one species); (iii) differences in pathogenicity of BYDVs. Results presented here confirmed BYDV-PAS infections, both in cultivated cereals and in perennial grasses. Moreover, based on the data of Strażyński and Roik [37], 3 of the main vectors of BYDV-PAS, *Ropalosiphum padi*, *Sitobion avenae,* and *Metopolophium dirhodum*, are still the most numerous species among the economically important group of aphids in Johnson’s suction trap in Poland. The obtained results may also be related to the fact that BYDV-PAS induces more severe symptoms than BYDV-PAV [28,32], and for this study, the samples with clear symptoms were collected. 

Comparative sequence analysis showed that the Polish BYDV-PAS isolates are highly similar to each other and share overall >98% and >99% nt similarity for RdRp and CP, respectively. However, a detailed analysis of the available 52 CP sequences and 33 RdRp sequences revealed some variability. The group of Polish isolates was more similar to the group of isolates from Europe and Morocco than to those from the USA, New Zealand, or Pakistan. In conclusion, similarly to Hall [38], current sequence alignment studies confirmed that the CP of BYDV-PAS was less diverse than RdRp. However, as it was previously reported for BYDV-PAV [21,29], sequence variability within two short 5′ CP hyper-variable domains, covering 45–60 and 155–168 aa residues, also affects the grouping of BYDV-PAS isolates. This division was reflected in phylogenetic relationships related rather to the mentioned genetic diversity than with geographic origin or hosts of studied isolates. As proof of this statement, cluster Ia grouped the isolates originated from different countries and infecting different host plants: BYDV-PAS-Imavere (Estonia, *Secale cereale*), BYDV-PAS-OH3 (the USA, *Triticum aestivum*), BYDV-PAS-2 (Germany, *Lolium multiflorum*); BYDV-P10 (Poland, *Bromus* sp.), BYDV-PAS-TR60-S51 (Turkey, *Geranium dissectum*) and BYDV-PAS-Pecq (Belgium, *Hordeum vulgare*). The findings concerning BYDV-PAS isolates did not confirm such close relationships between phylogeny, geographical affiliation, or host as it was presented for the BYDV-PAV population by Wu et al. [39].

Next, the presented findings confirmed that both South Korean BYDV-PAS-JE and BYDV-PAS-KJ isolates, same as previously characterized by Hodge et al. [33], American BYDV-PAS-OH2, were distinct from the others. The RdRp fragment of BYDV-PAS-JE, -KJ, and BYDV-PAS-OH2 shared, respectively, from 78% to 84.2% and from 86.6% to 87.6% aa sequence similarity with the others. Analogically, for CP, these values ranged from 72.2% to 86.1% and from 91% to 93.2%, respectively. So, both of their analyzed genome regions were more than 10% divergent at the aa level from other BYDV-PAS isolates. According to current rules of classification within the Luteoviridae family [24], they should be classified as novel variants of BYDV-PAS, like it was presented for BYDV-PAS-OH2 by Hodge et al. [33], or even as separate BYDV species. The distinctness of these isolates was also strongly supported by maximum likelihood phylogenetic trees generated using CP and RdRp nt sequences. As it was shown in the CP tree, listed above virus isolates did not cluster with either of 2 large clades of BYDV-PAS and created two distinct branches (BYDV-PAS-OH2 with BYDV-PAS-KJ and BYDV-PAS-JE) (Figure 6) while in RdRp tree BYDV-PAS-OH2 grouped alone and BYDV-PAS-KJ clustered together with BYDV-GAV which was used as an outgroup (Figure 7). Due to a confirmed recombination event in the BYDV-PAS-JE for RdRp fragment, it was rejected from the analyzed group of isolates because, as it was previously reported, recombination disturbs the proper classification of BYDV species [31]. Taking into account the great importance of recombination presence, such comparative studies using whole genome sequences of BYDV-PAS should be continued in the future. 

The present study is the first report providing molecular characteristics of the Polish isolates of BYDV-PAS and a current description of the phylogenetic relationships of its world’s population. Based on the obtained results, it can be assumed that this species is the most frequently occurring BYDV species in this region of Europe. Taking together this knowledge can contribute to understanding of BYDV epidemiology and will help to determine the changes in BYDV structure over time. 

## 4. Materials and Methods

### 4.1. Plant Sources

The plant samples of winter barley (30) and wheat (19) with stunting and leaf yellowing symptoms, as well as one symptomatic wild grass (*Bromus sterillis*), were collected from commercial fields in 20 various locations, in the spring of 2021 (from April to June). Samples originated from northern [Western-Pomerania (5), Pomerania (10), Kuyavia-Pomerania (2)], central [Lubusz (15), Greater Poland (17)] as well as southern [Subcarpathia (1)] regions of Poland. Additionally, the samples of wheat (1) and triticale (1) from Western Pomerania, wheat (1) from Warmia-Masuria, and barley (3) from Lower Silesia, collected in 2015 and 2018, were also used in this study.

### 4.2. RNA Isolation

Total RNA was extracted from all 56 symptomatic plant samples using the Total RNA Purification Kit (Novazym, Poznań, Poland) according to the manufacturer’s instructions. The concentration of total RNA was measured with a NanoDrop 2000 spectrophotometer (NanoDrop Technologies, Thermo Fisher Scientific, Waltham, MA, USA) and stored at −20 °C. 

### 4.3. Reverse Transcription-Polymerase Chain (RT-PCR)

The one-step RT-PCR reactions were used to amplify the sequences of BYDV CP (641 bp) and a portion of RdRp (955 bp) genes. The reactions were performed with the primers BYcp-F/BYcp-R designed by Kundu [30] and with PASrdrp-F (AGGACTTCATCAGCCACTGC) and PASrdrp-R (AAAAACCCTGGGAATCAAGC), developed for the purpose of this study. The oligonucleotides were designed by Primer3software (http://frodo.wi.mit.edu/, accessed on 11 September 2023) [40] based on the full-length nt of BYDV-PAS-Jogeva1 (MK012659). The reactions, for all isolated samples, were carried out using 0.2 µL of RevertAid Reverse Transcriptase 200U/µL (Thermo Fisher Scientific), 5 µL of Dream Taq Green PCR Master Mix (2X) (Thermo Fisher Scientific), 0.2 µL Primer Mix (10 µMol/µL each) and sterile Milli-Q water for a final volume of 10 µL. Amplification was performed in thermal conditions as follows: reverse transcriptase at 42 °C for 20 min, then initial denaturation at 94 °C for 3 min, 40 cycles of 30 s at 94 °C, 30 s at 55 °C for CP, and 50 °C for RdRp, 60 s at 72 °C and a final elongation at 72 °C for 5 min. Additionally, to detect and amplify CP of the WSMV genome (1000 bp), RT-PCR one-step reactions with specific WSMV-CPeur-F/WSMV-CP-R primers were performed according to the procedure presented by Trzmiel et al. [41]. The presence of specific RT-PCR products was verified on 1% TBE gel electrophoresis stained with Midori Green DNA Stain (Nippon Genetics Europe GmbH, Düren, Germany) for UV light visualization.

### 4.4. Restriction Fragment Length Polymorphism Analysis (RFLP)

In order to discriminate BYDV species, additional RFLP reactions were carried out according to Kundu et al. [34]. Amplified DNA samples (CP region) were digested by Hpa II endonuclease (Thermo Fisher Scientific) at 37 °C for 3 h and then separated in 2% TBE agarose gel and stained as described above.

### 4.5. Immuno-Capture-Duplex Polymerase Chain Reaction (IC-Duplex PCR)

In order to identify barley- or wheat-specific forms (WDV-B and WDV-W, respectively), IC-Duplex PCR was carried out. The reactions were performed with WDV-H-F/WDV-H-R and WDV-T-F/WDV-T-R primer pairs, according to the procedure described by Trzmiel [19]. Reaction results were checked by electrophoresis of obtaining products in 1% TBE agarose gel as described above. 

### 4.6. DNA Sequencing

Fourteen selected RT-PCR amplicons, obtained from barley, wheat, and *B. sterillis* plants originating from various regions of Poland were used for direct sequencing (Table 1). Reaction products of 955 and 641 pb in size, specific for RdRp and CP genes, respectively, were excised from agarose gels and purified using the Wizard^®^ SV Gel and PCR Clean-Up System (Promega Corp., Madison, WI, USA). Eluted DNA samples were subsequently sequenced by Genomed S.A. (Warsaw, Poland) using specific primers (PASrdrp-F/PASrdrp-R and BYcp-F/BYcp-R). The nucleotide sequences were analyzed using Standard Nucleotide BLAST (BLAST, http://blast.cnbi.nlm.nih.gov/Blast.cgi, accessed on 11 September 2023), compiled and edited using the BioEdit software, version 7.2.5 [42]. The nucleotide sequences of the RdRP fragment and CP complete genes were deposited in the National Center for Biotechnology Information (NCBI) GenBank database with accession numbers listed in Table 1. 

### 4.7. Estimating Sequence Similarity and Recombination Events

Partial genome sequences of 14 BYDV-PAS isolates obtained in this study and 47 BYDV-PAS isolates retrieved from the GenBank database (Table 1) were used in phylogenetic analyses. The alignments were performed for fragments of coding sequences of RdRp (ORF2) and CP (ORF3). The multiple sequence alignments were conducted using the ClustalW implemented in BioEdit program [42,43]. Sequence identity matrices were displayed using BioEdit and Sequence Demarcation Tool Version 1.2 (SDTv1.2) [44]. Phylogenetic analyses were preceded by an estimation of the occurrence of potential recombination events within the above-mentioned BYDV-PAS isolates. For this purpose, RDP, GENECONV, Chimaera, MaxChi, BootScan, SiScan, 3Seq, and LARD methods implemented in the Recombination Detection Program version 4 (RDP4) with default settings [45] were used. Recombination events were considered significant if four or more of these methods had a *p* < 0.05 in addition to phylogenetic evidence of recombination.

### 4.8. Phylogenetic Analysis

Phylogenetic studies were conducted for two amplified 942 and 535 nt trimmed fragments, respectively, for RdRp and CP genes within the 5′ and 3′ ends of the viral genome. Due to nt sequence identity a set of 6 different representative sequences for the CP gene of the Polish BYDV-PAS-P1, -P2, -P6, -P10, -P11, -P13 and 11 different sequences for RdRp gene of BYDV-PAS-P1, -P2, -P3, -P4, -P5, -P6, -P10, -P11, -P12, -P13, -P14 isolates, as well as 46 (for CP) and 22 (for RdRp) other BYDV-PAS sequences from the GenBank database, with deleted recombinant variants, were used for the analysis (Table 1). Chinese BYDV-GAV (AY220739) served as an outgroup. The phylogenetic relationships were analyzed by maximum likelihood algorithm (ML), choosing the best DNA/Protein model Kimura 2-parameter (K2+G) for nt and Jones-Taylor-Thornton (JTT+G) and Le Gascuel (LG+G) for deduced amino acids (aa) sequences, implemented in MEGA11 [46]. Bootstrap values were calculated using 1000 random replications. Phylogenetic trees were edited and visualized in the Evolview online platform [47].

## Figures and Tables

**Table 1:** Description of virus isolates used in this study.

Virus Isolate	Host	Origin	Acc. No. of RNA [CP * and RdRP #]
BYDV-PAS-P1 ^1^	*Triticale*	Poland, 2015	KU893144 *; ON950062 #
BYDV-PAS-P2	*Triticum aestivum*	Poland, 2015	KU893145 *; ON950063 #
BYDV-PAS-P3	*Hordeum vulgare*	Poland, 2015	ON950056 *; ON950064 #
BYDV-PAS-P4	*Triticum aestivum*	Poland, 2015	OM685192 *; ON950065 #
BYDV-PAS-P5	*Hordeum vulgare*	Poland, 2018	ON950057 *; ON950066 #
BYDV-PAS-P6	*Hordeum vulgare*	Poland, 2018	ON950058 *; ON950067 #
BYDV-PAS -IDA-P7	*Hordeum vulgare*	Poland, 2021	OM685192 *; ON950065 #
BYDV-PAS-P8	*Hordeum vulgare*	Poland, 2021	OM685192 *; ON950065 #
BYDV-PAS-P9	*Hordeum vulgare*	Poland, 2021	OM685192 *; ON950065 #
BYDV-PAS-Br-P10	*Bromus* sp.	Poland, 2021	ON109387 *; ON950068 #
BYDV-PAS-P11	*Hordeum vulgare*	Poland, 2021	ON950059 *; ON950069 #
BYDV-PAS-P12	*Hordeum vulgare*	Poland, 2021	OM685192 *; ON950070 #
BYDV-PAS-P13	*Hordeum vulgare*	Poland, 2021	ON950060 *; ON950071 #
BYDV-PAS-P14	*Triticum aestivum*	Poland, 2021	ON950061 *; ON950072 #
BYDV-PAS-Jogeva1 ^2^	*Triticum aestivum*	Estonia, 2015	MK012659
BYDV-PAS-Jogeva2	*Triticum aestivum*	Estonia, 2015	MK012658
BYDV-PAS-Jogeva4	*Triticum aestivum*	Estonia, 2015	MK012654
BYDV-PAS-Matapera	*Triticum aestivum*	Estonia, 2015	MK012657
BYDV-PAS-Vaimela	*Triticum aestivum*	Estonia, 2014	MK012655
BYDV-PAS-Puide	*Triticum aestivum*	Estonia, 2015	MK012656
BYDV-PAS-Olustvere2-W	*Triticum aestivum*	Estonia, 2014	MK012652
BYDV-PAS-Olustvere2-B	*Hordeum vulgare*	Estonia, 2014	MK012653
BYDV-PAS-Rannu1	*Triticum aestivum*	Estonia, 2014	MK012651
BYDV-PAS-Imavere	*Secale cereale*	Estonia, 2015	MK012660
BYDV-PAS-Polva	*Triticum aestivum*	Estonia, 2013	MK012650
BYDV-PAS-26-1	*Hordeum vulgare*	Germany, 2015	KY634888 *
BYDV-PAS-26-3	*Hordeum vulgare*	Germany, 2015	KY634890 *
BYDV-PAS-26-4	*Hordeum vulgare*	Germany, 2015	KY634891 *
BYDV-PAS-2	*Lolium multiflorum*	Germany, 2008	KY634879 *
BYDV-PAS-131	*Triticum aestivum*	Germany, 2015	KY634913 *
BYDV-PAS-138	*Festuca ovina*	Germany, 2008	KY634917 *
BYDV-PAS-114	*Hordeum vulgare*	Germany, 2015	KY634907 *
BYDV-PAS-143-1	*Hordeum vulgare*	Germany, 2016	KY634922 *
BYDV-PAS-142-3	*Hordeum vulgare*	Germany, 2016	KY634921 *
BYDV-PAS-Pecq	*Hordeum vulgare*	Belgium, 2013	OM046619 *
BYDV-PAS-TR60-S51	*Geranium dissectum*	Turkey, 2018	MZ612011
BYDV-MA9505	*-*	Morocco, 1998	AJ007920 *
BYDV-MA9511	*-*	Morocco, 1998	AJ007922 *
BYDV-MA9516	*-*	Morocco, 1998	AJ007926 *
BYDV-PAS-0109	*Hordeum vulgare*	USA: Iowa, 2007	EF521828
BYDV-PAS-OH1	*Triticum aestivum*	USA: Ohio, 2016	MN128938 *
BYDV-PAS-OH1A	*Triticum aestivum*	USA: Ohio, 2016	MK913614 *
BYDV-PAS-OH1B	*Triticum aestivum*	USA: Ohio, 2016	MK913615 *
BYDV-PAS-OH1D	*Triticum aestivum*	USA: Ohio, 2016	MK913617 *
BYDV-PAS-OH2	*Triticum aestivum*	USA: Ohio, 2016	MN128939
BYDV-PAS-OH3	*Triticum aestivum*	USA: Ohio, 2016	MN128940
BYDV-PAS-KS PAS-1	*Triticum aestivum*	USA: Kansas, 2012	KY593456
BYDV-PAS-KS PAS-2	*Triticum aestivum*	USA: Kansas, 2012	KY593457
BYDV-PAS-KS-SHKR	*Triticum aestivum*	USA: Kansas, 2011	KU170668
BYDV-PAS-WG13	*-*	USA, 2005	DQ285676 *
BYDV-PAS-64	*-*	USA, 2005	DQ285679 *
BYDV-PAS-WF12	*-*	USA, 2005	DQ285678 *
BYDV-PAS-WS6603	*-*	USA, 2005	DQ285680 *
BYDV-PAS-129	*-*	USA, 2005	AF218798
BYDV-PAS37P5o03-2	*Avena sativa*	USA, 2003	DQ792506 *
BYDV-PAS-M40-2	*Pearl millet*	Pakistan, 2011	KR259156 *
BYDV-PAS-waw5-1	*Microlaena stipoides*	New Zealand	EF408152 *
BYDV-PAS-waw5-9	*Microlaena stipoides*	New Zealand	EF408153 *
BYDV-PAS-KJ	*Avena sativa*	Republic of Korea, 2020	LC592173
BYDV-PAS-JE	*Avena sativa*	Republic of Korea, 2020	LC592174
BYDV-PAS-CNU-GNW	*Triticum aestivum*	Republic of Korea, 2021	LC746087 *
BYDV-GAV	-	China	AY220739

^1^ local BYDV isolates. ^2^ BYDV isolates retrieved from the GenBank database. * CP gene sequence accession number. # RdRp gene sequence accession number.

## Data Availability

The datasets generated during the current study are available from the corresponding author upon reasonable request.
